# Prevalence of osteoporosis in patients with nephrolithiasis and *vice versa*: a cumulative analysis

**DOI:** 10.3389/fendo.2023.1180183

**Published:** 2023-07-04

**Authors:** Shunjie Jia, Jian Liao, Yucheng Wang, Wenbiao Zheng, Jinghua Jin, Weifang Xu, Qi Zheng

**Affiliations:** ^1^ Department of Orthopedics, Taizhou Municipal Hospital, Taizhou, Zhejiang, China; ^2^ Department of Nephrology, Jiaxing Hospital of Traditional Chinese Medicine, Jiaxing, Zhejiang, China; ^3^ Department of Orthopedics, Taizhou Central Hospital (Taizhou University Hospital), Taizhou, Zhejiang, China

**Keywords:** osteoporosis, nephrolithiasis, prevalence, risk, cumulative analysis

## Abstract

**Purpose:**

Nephrolithiasis is thought to be a risk factor for osteoporosis, but data assessing if osteoporosis predisposes to the risk of nephrolithiasis are lacking. The present study aims to investigate whether patients with nephrolithiasis have a prominently higher prevalence of osteoporosis than the controls and *vice versa* via a cumulative analysis.

**Methods:**

Four databases were used to detect the eligible studies. We calculated the relative risk (RR) with a 95% confidence interval (CI) to assess the combined effect. The methodologies for conducting this study followed the PRISMA guidelines and were registered in the PROSPERO (ID: CRD42023395875),

**Results:**

Nine case-control or cohort studies with a total of 454,464 participants were finally included. Combined results indicated that there was a significantly higher prevalence of osteoporosis in patients with nephrolithiasis as compared to the general population without nephrolithiasis (overall RR from six studies= 1.204, 95%CI: 1.133 to 1.28, *P*< 0.001; heterogeneity: *I^2 ^= *34.8%, *P*= 0.162). Conversely, osteoporosis was significantly correlated to an increased risk of nephrolithiasis as compared to the controls without osteoporosis (overall RR from four studies= 1.505, 95%CI: 1.309 to 1.731, *P*< 0.001; *I^2 ^= *89.8%, *P*< 0.001). Sensitivity analysis on the two categories validated the above findings. No significant publication bias was identified in this study.

**Conclusions:**

The present study highlighted a significantly high prevalence of osteoporosis in patients with nephrolithiasis and *vice versa*. This reciprocal association reminded the clinicians to conduct a regular follow-up assessment when managing patients with nephrolithiasis or osteoporosis, especially for the elderly.

**Systematic review registration:**

https://www.crd.york.ac.uk/PROSPERO/#searchadvanced, identifier CRD42023395875.

## Introduction

Nephrolithiasis, a common urological disorder, constitutes an important health concern and a common cause of pain and hospitalization. The incidence of nephrolithiasis ranges from 7–13%, 5–9%, and 1–5%, in North America, Europe, and Asia, respectively (1). Various factors such as geography, climate, diet, fluid intake, genetics, gender, occupation, and age can affect the rate of nephrolithiasis (2–4). Without timely treatment, nephrolithiasis may cause serious complications, e.g., infection, nephrorrhagia, hydronephrosis, kidney injury, and even renal failure (5). It was previously reported that $2.1 billion were cost on the medical management of urolithiasis in the United States in 2000 (6), while this fee rose to $5 billion per year by 2030 (7). Nephrolithiasis is a multifactorial disease. Its formation mechanisms have not yet been elucidated. Hypercalciuria is considered to serve as a common etiopathogenesis for nephrolithiasis formation (8). According to several reports, nephrolithiasis formation is correlated with an increased risk of developing hypertension, diabetes mellitus, gallstones, renal cell carcinoma, and transitional cell carcinoma of the upper urinary tract (9, 10). Since both nephrolithiasis and osteoporosis are metabolic diseases, mounting evidence indicates that nephrolithiasis is reciprocally linked to osteoporosis (11). Calcium is the main calculus component, nearly 80% of nephrolithiasis is composed of calcium oxalate (12). It was found that nephrolithiasis patients are liable to suffer from an increased rate of bone resorption and a lower bone mineral content, especially in those with idiopathic calcium stones (12). Therefore, nephrolithiasis may be a key risk factor for osteoporosis.

Osteoporosis is a chronic, metabolic bone disorder in the elderly, manifested by the deterioration of bone mineral density (BMD) and impaired bone micro-architecture (13). It affects about 10% of individuals aged > 50 and up to about 25% of those aged over 80. Osteoporosis is associated with an increased risk of bone fracture, causing disability and mortality of the sufferers (14). Since the incidence of both nephrolithiasis and osteoporosis increases with age and both of them are metabolic disorders, thus it is expected that they may share a common pathogenesis. The pathogenic mechanisms underlying these two diseases may be associated with the intensive interaction between genetic and environmental factors (11). Besides, the natural histories of the two diseases have many similarities, such as morbidity, the characteristics of the disease course, and the adverse consequences without proper management. Based on these findings, a previous meta-analysis (15) summarized the early evidence and demonstrated that nephrolithiasis was associated with dramatically lower values of BMD T-scores in the spine, total hip, and femoral neck. In addition, this meta-analysis also pooled the results from two relevant studies and found that patients with nephrolithiasis have a higher prevalence of osteoporosis than the healthy controls without nephrolithiasis (odds ratio = 4.12, 95% confidence interval: 3.99 to 4.26, *P* < 0.0001) (15). Interestingly, the investigators have found not only a high prevalence of osteoporosis in nephrolithiasis patients but also a high prevalence of nephrolithiasis in osteoporosis patients, as compared to the controls. Keller et al. (16) showed that patients with osteoporosis were more likely to diagnose with nephrolithiasis when compared to controls (odds ratio=1.66, 95% confidence interval: 1.59 to 1.73, *P* < 0.05) after adjusting for multiple confounding factors.

Recently, there have been mounting trials demonstrating the reciprocal association between nephrolithiasis and osteoporosis. In the present study, we aimed to quantitatively summarize the current evidence from all the relevant published studies to evaluate the categorical association between nephrolithiasis and osteoporosis.

## Methods

This cumulative analysis was registered on the PROSPERO (ID: CRD42023395875), an international database of prospectively registered systematic reviews. Moreover, we followed the Preferred Reporting Items for Systematic Reviews and Meta-Analyses (PRISMA) guidelines when conducting this cumulative analysis (**Supplementary Table 1**).

### Data sources and searches

To identify the eligible studies, we searched four electronic databases, including MEDLINE (PubMed), the Cochrane Library databases, EMBASE (OVID), and PsychINFO, from their inception until December 1, 2022. Among the studies we included, only those involving human participants reported using English. PubMed search keywords with various combinations were as follows: ((((((((((((((((((((((“Osteoporosis”[Mesh]) OR (Osteoporoses)) OR (Osteoporosis, Post-Traumatic)) OR (Osteoporosis, Post Traumatic)) OR (Post-Traumatic Osteoporoses)) OR (Post-Traumatic Osteoporosis)) OR (Osteoporosis, Senile)) OR (Osteoporoses, Senile)) OR (Senile Osteoporoses)) OR (Osteoporosis, Involutional)) OR (Senile Osteoporosis)) OR (Osteoporosis, Age-Related)) OR (Osteoporosis, Age Related)) OR (Bone Loss, Age-Related)) OR (Age-Related Bone Loss)) OR (Age-Related Bone Losses)) OR (Bone Loss, Age Related)) OR (Bone Losses, Age-Related)) OR (Age-Related Osteoporosis)) OR (Age Related Osteoporosis)) OR (Age-Related Osteoporoses)) OR (Osteoporoses, Age-Related)) AND (((((((((((((((((“Urolithiasis”[Mesh]) OR (Urinary Lithiasis)) OR (Lithiasis, Urinary)) OR (Kidney Calculi)) OR (Calculi, Kidney)) OR (Calculus, Kidney)) OR (Kidney Calculus)) OR (Nephrolith)) OR (Renal Calculus)) OR (Kidney Stones)) OR (Kidney Stone)) OR (Stone, Kidney)) OR (Stones, Kidney)) OR (Renal Calculi)) OR (Calculi, Renal)) OR (Calculus, Renal)) OR (Nephrolithiasis)).

### Measurement of osteoporosis and nephrolithiasis

The BMD T-score criteria are commonly used to diagnose osteoporosis. A T-score ≤ −2.5 in any of the bones was applied for defined osteoporosis. Nephrolithiasis could be confirmed by ultrasound and radiograph examination (i.e., X-rays, computerized tomography).

### Inclusion criteria

This cumulative analysis included all epidemiologic studies that met the prior inclusion criteria. The question guiding the present study was: Does nephrolithiasis increase the risk of osteoporosis and *vice versa*? The Patient, Intervention, Comparison, Outcome, and Study Design (PICOS) framework was applied. The components for this PICOS evidence contained the following factors: patients diagnosed with osteoporosis or nephrolithiasis (P); a history of nephrolithiasis or osteoporosis (I); compared with the healthy controls (C); the prevalence of osteoporosis or nephrolithiasis (O); any study designs (S). Furthermore, those articles that provided relative risk (RR) or odds ratios (OR) with the corresponding 95% confidence intervals (CI) were also considered to be eligible.

### Exclusion criteria

The exclusion criteria of this cumulative analysis were: (a) non-English studies, (b) lack of a control group; (c) review articles, comments, and case reports; (d) duplicated data; (e) experimental studies (i.e., *in vivo* or *in vitro* studies); (f) meta-analysis researches. Under the inclusion and exclusion criteria, two authors independently screened the potential included studies. The corresponding author or the third author was expected to resolve the ambiguities during the selection of the eligible studies.

### Data extraction

By using a standardized data collection table, two authors independently evaluated and extracted the data, including the names of the first author, the publication year of the included studies, study location, study design, age of the participants, the number of cases of osteoporosis or nephrolithiasis in both the study and the control group, the RR accompanied with the 95%CI generated in each included study, and the assessments of osteoporosis or nephrolithiasis ascertainment.

### Quality assessment

Two independent authors assessed the methodological quality of the eligible studies. Any ambiguities were resolved by consensus or the third author. The methodological quality of case-control and cohort studies was followed by the Newcastle–Ottawa Scale (NOS). Within this scale, nine domains were assessed and the score of conformity gained one score. Studies were assigned a score between 0 and 3 to indicate low quality, 4 to 6 to indicate moderate quality, and 7 to 9 to indicate high quality.

### Statistical analyses

We conducted the current cumulative analysis by using the STATA (version 13.0, Stata Corp LP, College Station, Texas, USA). The combined RR with the corresponding 95% CI was applied to quantitatively evaluate the strength of the association between nephrolithiasis and osteoporosis. Two-tail P values of 0.05 were assumed to be statistically significant. The heterogeneity test was conducted using *I* ^2^ statistics and the Cochrane Q statistic. Significant heterogeneity was defined as *I* ^2^ > 50%. In the Q test, a *P*-value < 0.10 is considered statistically significant. Considering the high likelihood of between-study variance for differences in the study design and demographics, a random-effect model is used in this cumulative analysis rather than a fixed-effect model. Additionally, we performed a sensitivity analysis to investigate the sources of heterogeneity. In sensitivity analyses, one study was eliminated at a time and the effect of this elimination was subsequently evaluated. An assessment of publication bias was conducted using the funnel plot and Begg’s rank correlation test.

## Results

### Literature search

**Figure 1:**
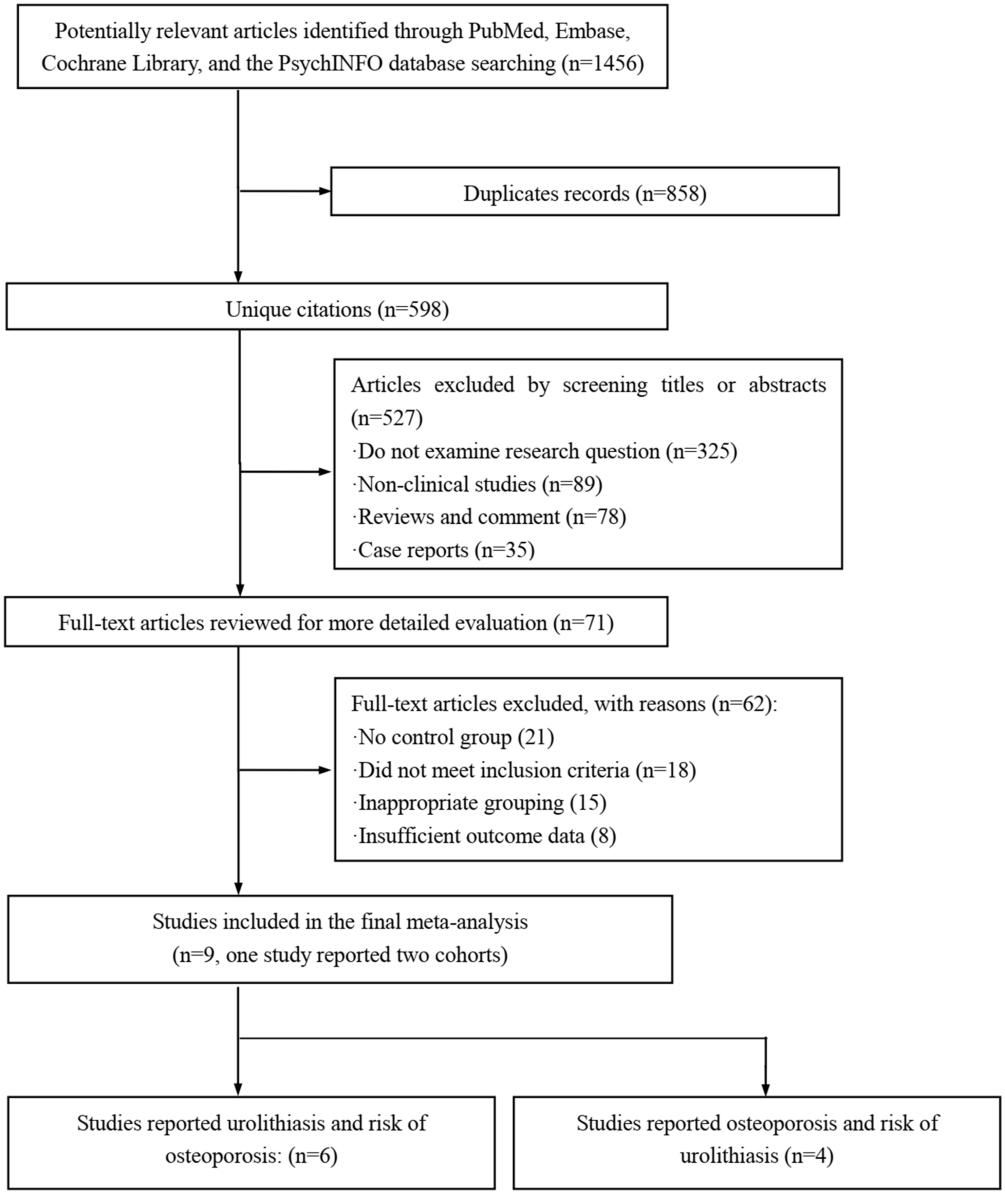
Flow chart of study selection.

The selection process for screening the potential inclusions is shown in **Figure 1**. A total of 1456 publications were screened during the initial search within the databases of MEDLINE, Cochrane Library databases, EMBASE, and PsychINFO. There were 71 articles were retrieved for full-text review after eliminating duplicates and those studies out of the aforementioned inclusion criteria. Among the 71 potential trials, 21 studies were excluded due to without a control group; 18 studies did not meet the inclusion criteria; 15 studies for inappropriate grouping; 8 studies for insufficient outcome data. Finally, nine studies (16–24) were included in this cumulative analysis. Among these eligible studies, one study conducted by Kim et al. (22) provided the prevalence of osteoporosis in nephrolithiasis patients as well as the prevalence of nephrolithiasis in patients with osteoporosis. So, six eligible studies reported nephrolithiasis and the risk of osteoporosis and four eligible studies reported osteoporosis and the risk of nephrolithiasis were conducted for synthesizing the overall RR for the two topics.

### Study characteristic

**Table 1** showed the characteristics of the six included studies of nephrolithiasis and risk of osteoporosis and four included studies of osteoporosis and the risk of nephrolithiasis (one study provided two independent data). Among the nine included studies, five of them were designed for case-control and four studies were cohort design. These studies were published between 2002 and 2022. In terms of geographical distribution, five studies were developed in Europe and four studies were conducted in Asia. These studies involved participants ranging in mean age from 39.2 to 66.7. The sample size ranged from 64 to 135,622. The confirmation of osteoporosis mainly depended on the T-score and International Classification of Disease (ICD), while nephrolithiasis was diagnosed by ultrasound and radiograph, etc.

**Table 1:** Characteristics of the included studies.

Study	Study design	Mean age (years)	Study group case/total	Control group case/total	RR (95%CI)	Assessment of osteoporosis
Six included studies reporting the association between nephrolithiasis and risk of osteoporosis:						
Cvijetic (14) 2002 Croatia	Case- control	S: 42.3 ± 6.5 C: 39.2 ± 6.3	1/34	0/30	(1.67, 4.82)	T-score ≤ −2.5.
Arrabal-Polo (15) 2012 Spain	Case- control	S: 45.9 ± 12.1 C: 48.9 ± 10.1	Hip: 2/59 Spine: 9/59	Hip: 1/56 Spine: 2/56	Hip: 1.45 (1.29, 1.62) Spine: 1.45 (1.29, 1.62)	T-score ≤ −2.5.
Bijelic (16) 2014 Bosnia and Herzegovina	Case- control	S: 50.2 ± 15.6 C: 48.7 ± 17.0	9/120	1/120	1.45 (1.29, 1.62)	The instrument LUNAR DPX Product Division Americus GE Healthcare
Shavit (17) 2015 UK	Case- control	S: 47 ± 14 C: 47 ± 13	16/51	9/54	1.45 (1.29, 1.62)	BMD with CT attenuation in Hounsfield units (HU).
Lu (18) 2020 Chinese Taipei	Cohort	S: 59.7 ± 8.3 C: 59.8 ± 8.4	2884/22575	7438/68679	(1.67, 4.82)	T-score ≤ −2.5.
Kim (19) 2022 Korea	Cohort	NA	2319/25261	7658/101044	(1.67, 4.82)	ICD-10 codes: M80–M82; Bone density testing using X-ray or CT
Four included studies reporting the association between osteoporosis and risk of nephrolithiasis:						
Keller (13) 2013 Chinese Taipei	Case- control	S: 65.1 C: 64.9	11382/39840	7050/39840	(1.29, 1.62)	T-score ≤ −2.5.
Chou (20) 2014 Chinese Taipei	Cohort	≥ 50	60/1634	165/6536	(0.59, 1.65)	ICD-9-CM codes 733.X; BMD test
Rendina (21) 2021 Italy	Cohort	66.7 ± 10.7	447/10157	69/2637	(1.29, 1.62)	T-score ≤ −2.5.
Kim (19) 2022 Korea	Cohort	NA	2276/67811	1696/67811	(1.67, 4.82)	ICD-10 codes: M80–M82; Bone density testing using X-ray or CT

S, Study group, Patients with osteoporosis or urolithiasis; C, Control group, Healthy controls without osteoporosis or urolithiasis; RR, Relative risk; CI, Confidence interval; BMD, Bone mineral density; ICD, International Classification of Disease; NA, not available.

### Study methodological quality

Following NOS’ methodological quality checker, six included studies gained a score of 7-9, so they were considered high quality. The remainder three included studies were judged to a score of 6, indicating these studies were of moderate quality. As a result, the proportion of high methodological quality in this cumulative analysis was 67% (6/9). The detail-specific scoring was listed in **Supplementary Table 2**.

### Meta-analyses

**Figure 2:**
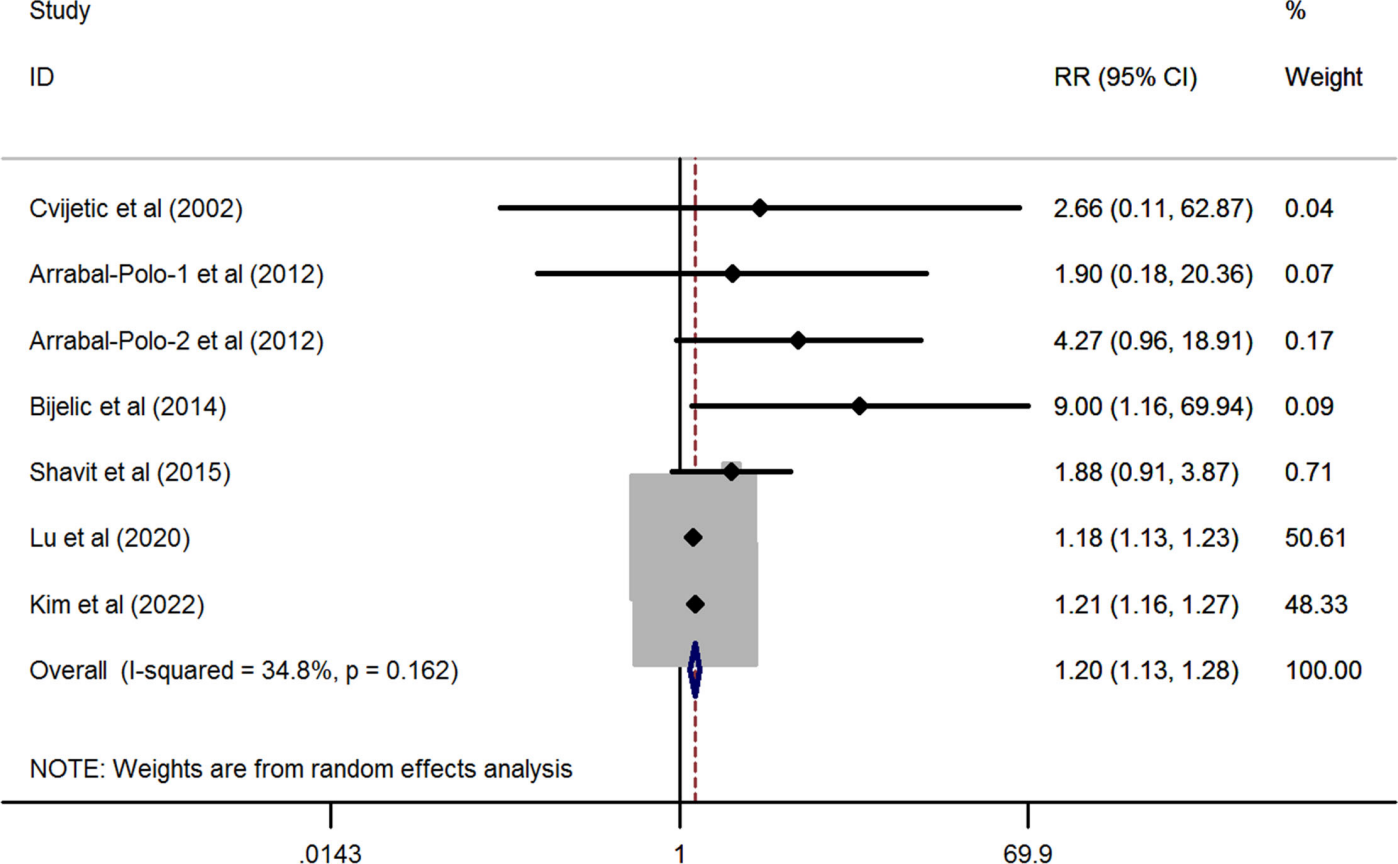
Forest plots of the cumulative analysis of the six included studies on the association between nephrolithiasis and the risk of osteoporosis.

As shown in **Figure 2**, the pooled result derived from six eligible studies (one study provided both the hip and spine data) demonstrated that there was a significantly higher prevalence of osteoporosis in patients with nephrolithiasis as compared to the general population without nephrolithiasis by using a random-effects model (overall RR from 7 studies= 1.204, 95%CI: 1.133 to 1.28, *P*< 0.001; heterogeneity test: *I^2 ^= *34.8%, *P*= 0.162).

**Figure 3:**
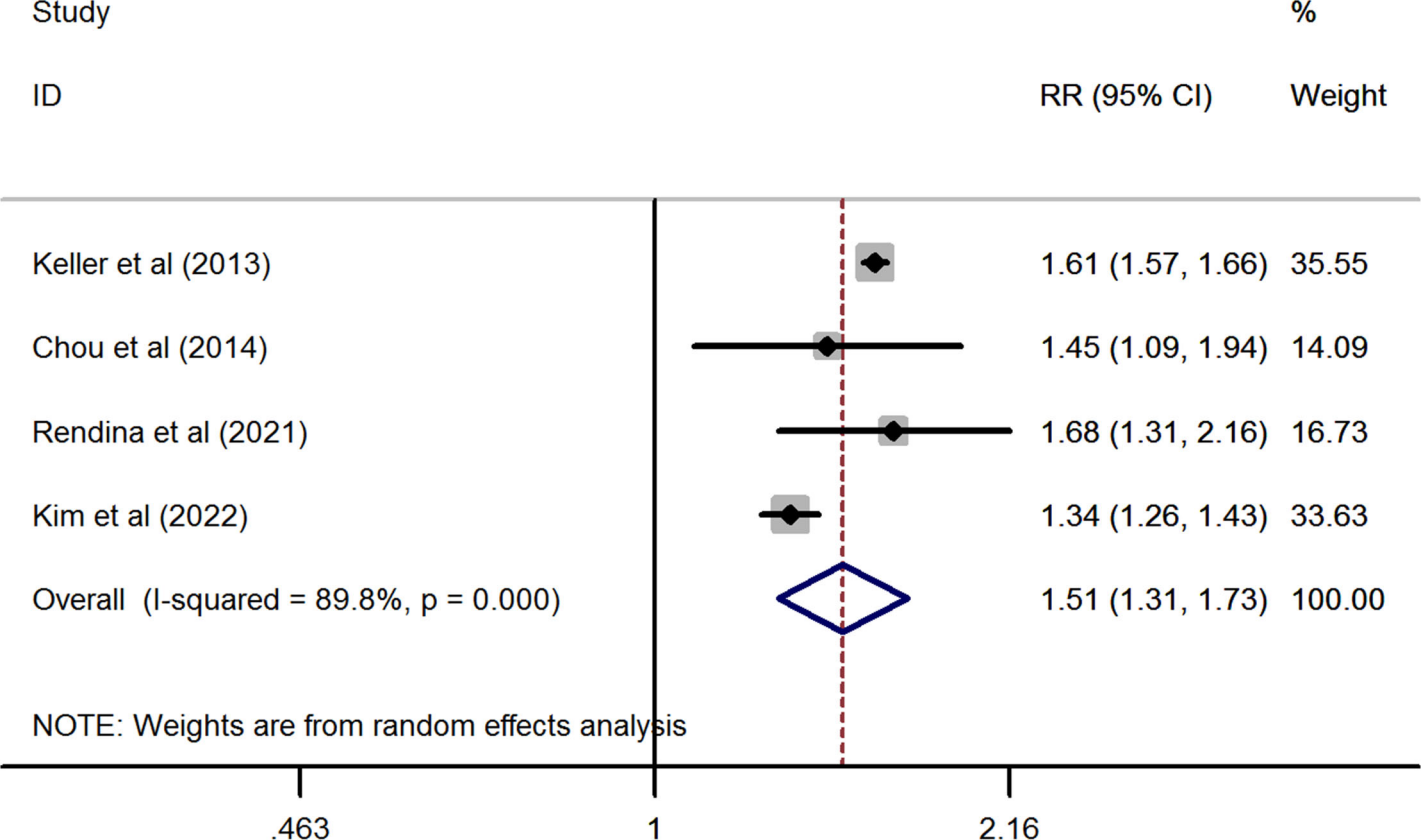
Forest plots of the cumulative analysis of the four included studies on the association between osteoporosis and the risk of nephrolithiasis.

In line with the above findings, the prevalence of nephrolithiasis was also significantly higher in patients with osteoporosis than in the healthy control without osteoporosis (pooled RR from four studies = 1.505, 95%CI: 1.309 to 1.731, *P*< 0.001). However, substantial heterogeneity was observed in this synthesized analysis (*I^2 ^= *89.8%, *P*< 0.001) (**Figure 3**).

The above results suggested that there was a cross-correlation between nephrolithiasis and osteoporosis. Nephrolithiasis might elevate the risk of osteoporosis. Conversely, patients with osteoporosis might also be susceptible to suffering from nephrolithiasis.

### Sensitivity analysis

**Figure 4:**
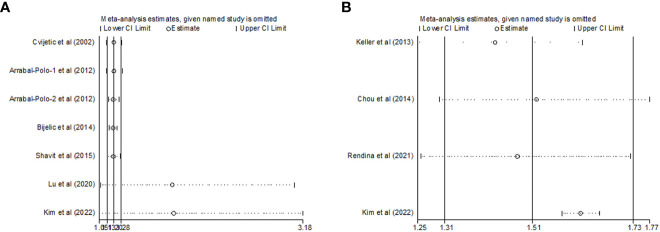
Sensitivity analysis after each study was excluded by turns in the categories **(A)** nephrolithiasis and risk of osteoporosis; **(B)** osteoporosis and risk of nephrolithiasis).

To evaluate the impact of individual studies on the overall RR, sensitivity analysis was conducted on the two topics. First, as shown in **Table 2**; **Figure 4A**, no significant change in the newly generated pooled RR was observed for studies that reported nephrolithiasis and the risk of osteoporosis. The RR ranged from 1.815 (95%CI: 1.067-3.184, *P*= 0.028) to 1.206 (95%CI: 1.128-1.289, *P*< 0.001). On the other hand, no substantial heterogeneity was observed after eliminating any one of the 7 included studies. The *I^2^* ranged from 8.8% to 44.8% and all *P*>0.1.

**Table 2:** Sensitivity analysis after each study was excluded by turns in the included studies.

Study omitted	RR (95% CI) for remainders	Heterogeneity		
		*I*2	*P*	
Nephrolithiasis and risk of osteoporosis				
Cvijetic et al. (2002)	1.206 (1.128, 1.288) *P*<0.001	44.2%	0.111
Arrabal-Polo-1 et al. (2012)	1.206 (1.128, 1.289) *P*<0.001	44.8%	0.107
Arrabal-Polo-2 et al. (2012)	1.199 (1.145, 1.255) *P*<0.001	21.8%	0.270
Bijelic et al. (2014)	1.197 (1.154, 1.241) *P*<0.001	8.8%	0.360
Shavit et al. (2015)	1.200 (1.133, 1.271) *P*<0.001	35.0%	0.174
Lu et al. (2020)	1.815 (1.067, 3.087) *P*=0.028	39.1%	0.145
Kim et al. (2022)	1.830 (1.051, 3.184) *P*=0.033	42.1%	0.124
Osteoporosis and risk of nephrolithiasis				
Keller et al. (2013)	1.422 (1.249, 1.618) *P*<0.001	36.6%	0.207
Chou et al. (2014)	1.515 (1.297, 1.769) *P*<0.001	93.2%	<0.001
Rendina et al. (2021)	1.472 (1.256, 1.725) *P*<0.001	93.2%	<0.001
Kim et al. (2022)	1.614 (1.572, 1.656) *P*<0.001	0.0%	0.742

RR, Relative risk; CI, Confidence interval.

As regards to those studies reporting osteoporosis and the risk of nephrolithiasis, there was no substantial change in the new synthesized overall RR, which ranged from 1.422 (95%CI: 1.249-1.618, *P*< 0.001) to 1.614 (95%CI: 1.572-1.656, *P*< 0.001) after eliminating any of the four included studies. However, the substantial heterogeneity remarkably disappeared after excluding Kim et al.’s study (22) (*I^2 ^= *0.0%, *P*= 0.742) (**Table 2**; **Figure 4B**). These results demonstrated that Kim et al.’s study might be the source of the great heterogeneity (*I^2 ^= *89.8%) in this cumulative analysis.

### Publication bias

**Figure 5:**
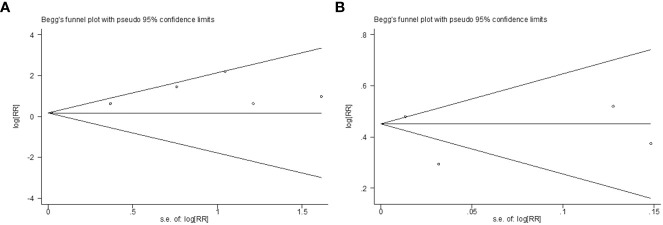
**(A)** Begg’s test for detecting publication bias on the topic of nephrolithiasis and risk of osteoporosis; **(B)** Begg’s test for detecting publication bias on the topic of and osteoporosis risk of nephrolithiasis.

According to Begg’s rank-correlation test, there was no significant publication bias in the seven studies reporting the association between nephrolithiasis and the risk of osteoporosis (Begg’s, *P* > |z| = 0.548) (**Figure 5A**). Consistent with this finding, Begg’s rank-correlation test also revealed no significant publication bias within the analysis of the relationship between osteoporosis and the risk of nephrolithiasis (Begg’s, *P* > |z| = 0.734) (**Figure 5B**).

## Discussion

Approximately 40 years ago, an earlier report conducted by Fuss et al. (25) demonstrated that a significant decreased bone mineral content (BMC) was observed in renal stone formers as compared to the normal (*P*<0.001). During the last 40 years, a considerable proportion of investigators found a reduction in both BMC and BMD in patients with calcium nephrolithiasis (26–29). In these studies, the prevalence of osteoporosis in stone-forming patients ranged from 6 to 60% (30). In a more recent study developed by Ganesan et al. in 2021, the authors reported that 20% of the 531,431 patients with kidney stone disease were subsequently diagnosed with osteoporosis (31). However, most of the above studies have a key limitation which is the lack of a control group. On the other hand, more and more authors have described that patients with osteoporosis are susceptible to nephrolithiasis (16, 19). Therefore, it would seem likely a reciprocal relationship exists between osteoporosis and nephrolithiasis. To better explore the association between nephrolithiasis and osteoporosis via a scientific method, we conducted this cumulative analysis focused on the two research questions. As a result of comprehensive searches for the included studies, six observational trials reporting nephrolithiasis and the risk of osteoporosis and three retrospective studies reporting osteoporosis and the risk of nephrolithiasis were eventually included in this pooled analysis. Synthetic RR from seven studies described a 1.2-fold elevated risk of osteoporosis in patients with nephrolithiasis compared to the controls without nephrolithiasis (a total of 218,198 participants, overall RR= 1.2, 95%CI: 1.13 to 1.28, *P*< 0.001). On the other hand, combined RR from four studies reporting the relationship between osteoporosis and risk of nephrolithiasis also yielded a 1.51-fold increased risk of nephrolithiasis in patients with osteoporosis as compared to the healthy population without osteoporosis (a total of 236,266 participants, overall RR= 1.51, 95%CI: 1.31 to 1.73, *P*< 0.001). In this pooled analysis, the methodological quality of the nine included studies was either moderate or high quality. And the proportion of high methodological quality among the eligible studies was up to 67%. Besides, sensitivity analysis suggested that no single study dominated the overall RR in the two independent cumulative analyses. Moreover, no remarkable publication bias was detected when combining the RR from the included studies. Based on the above facts, our study is highly reliable and the evidence is robust.

Though there has been a positive association between nephrolithiasis and osteoporosis found in the current cumulative analysis and other uncontrolled studies, a clear etiology for such a correlation is still elusive. Theoretically, both nephrolithiasis and osteoporosis are calcium metabolic disorders, indicating there are some commonalities between the two diseases. According to the findings from some relevant studies, the predominant etiological agents underlying the relationship between nephrolithiasis and osteoporosis might be hypercalciuria and dietary calcium intake (15).

It is known that patients with hypercalciuria may be predisposed to bone demineralization (32). It has been reported that 20 to 30% of patients initially diagnosed with osteoporosis suffer from idiopathic hypercalciuria, a subclinical condition that contributes most to bone loss (15, 33, 34). The pathogeny of the loss of BMD in patients with idiopathic hypercalciuria is not well-understood. Hypercalciuria has been described to correlate to calcium-phosphate homeostasis, promotion of cytokines predisposing to bone loss, the elevation of parathormone, and calcitriol-mediated cascades. Hypercalciuria can alter the calcium-phosphate homeostasis of the body. In patients with hypercalciuria, this condition may cause tubular dysfunction of the Na-P cotransporters, which is associated with renal phosphate leak and thus predisposing to bone demineralization (35). Patients with hypercalciuria are susceptible to suffering from the disorders of calcium–phosphate and vitamin D homeostasis. Since vitamin D or calcium is essential for bone health, osteoporosis may be attributable to deficiencies in these two nutrients (36). Hypercalciuria was found to be associated with low serum vitamin D levels (37), while low vitamin D significantly influenced bone loss. Vitamin D functions to increase the intestinal absorption of calcium and phosphorous. Also, Lacey et al. (38) reported that vitamin D could regulate IL-1 and IL-6 production and thus affect bone metabolism in a dose-dependent way. As a result, adequate calcium and vitamin D intakes were considered to be beneficial for osteoporosis (39). On the other hand, however, calcium and vitamin D supplementations are risk factors for stone formation. Therefore, patients who received calcium and vitamin D treatment may induce nephrolithiasis (36). It showed a 17% elevated risk of nephrolithiasis in patients treated with calcium and vitamin D (40). But this speculation did not support by some investigators. Penniston et al. (41) found that vitamin D supplements did not elevate urine calcium and the risk of nephrolithiasis. Similar to this study, Arrabal-Polo et al. (42) demonstrated that patients with nephrolithiasis presented higher calcium excretion in 24-hour urine, irrespective of vitamin D excretion rates. In terms of genetic determinants, a high level of intestinal vitamin D receptors was found in the hypercalciuric animal model (43). It was reported that BMD could be regulated by a simple allelic substitution in the vitamin D receptor gene (44). And the vitamin D receptor polymorphisms were found to be associated with calcium kidney stone disease (12). Some investigators found an increase in vitamin D receptors’ sensitivity affecting bone resorption (12, 30), indicating a role of vitamin D in the development of osteoporosis. In addition to hypercalciuria, hyperuricosuria may also play a role in the relationship between nephrolithiasis and osteoporosis. Hyperuricosuria was found to not only promote the formation of calcium oxalate stones but also associated with lower BMD (17, 45, 46). Though the relationship among hypercalciuria, vitamin D, and bone health is still unclear, several studies pointed out that thiazides might be more beneficial than calcium and vitamin D supplements in treating patients with osteoporosis and hypercalciuria (47, 48). The underlying mechanisms that might be correlated to thiazides can elevate renal tubular reabsorption of calcium and increase the levels of serum calcium, thus reducing lithogenic risk as well as improving BMD (49).

In addition to vitamin D, intact parathyroid hormone (PTH) is also one of the pivotal hormones responsible for the metabolism of calcium and phosphorous. A parathyroid-independent pathologic process may cause bone Ca efflux. Excess PTH levels induce additional loss of calcium in the sufferers, which leads to the formation of calcium stones (50, 51). In individuals with hyperparathyroidism, urinary tract stones, and vertebral fractures occur frequently (52). Higher PTH levels may decline the circulating 25-hydroxyvitamin D by facilitating renal conversion to 1,25-dihydroxyvitamin D (53). A surplus of PTH was found to induce calcium loss with a consequential creation of calcium stones as well as lessen BMD and develop osteopenia and osteoporosis (54). Interestingly, Reid et al. (53) showed that the occurrence of osteoporosis was high in hyperparathyroidism patients, but the rate of nephrolithiasis was not high in these patients. This finding suggested that PTH disturbance-mediated osteoporosis might not be relevant to nephrolithiasis development. Thus, the potential role of PTH in the correlation between nephrolithiasis and osteoporosis needs prospective evaluation.

Alternatively, the diet has also been postulated to be an important factor underlying the correlation between osteoporosis and nephrolithiasis. Theoretically, nephrolithiasis patients may restrict calcium and vitamin D intake to prevent the recurrence of urinary tract stones, and this may contribute to the development of osteoporosis (55). Limited calcium intake may cause a negative calcium balance that increases the risk of bone loss and BMD depletion over time. Conversely, patients with osteoporosis are more likely to be treated with calcium and vitamin D, which may increase the risk of renal stone formation. This is due to vitamin D, calcium, or combined supplementation was found to be associated with an increase in the incidence of kidney stones (36). Calcium dietary intake was previously found to promote oxalate absorption and excretion. Of note, however, many investigators observed that both vitamin D and calcium supplementation did not elevate the risk of nephrolithiasis (56, 57). In addition to hypercalciuria, both nephrolithiasis and osteoporosis may share other common pathogenic factors, e.g. unhealthy eating habits. It was reported that patients with nephrolithiasis or osteoporosis tended to have high salt, high sugar intake, and low physical activity (11, 58, 59). Nouvenne et al. (60) revealed that high sodium in the diet might elevate the risk of nephrolithiasis and osteoporosis on account of a higher urinary calcium excretion and lower citrate excretion. However, diets higher in potassium intake are inversely correlated with both nephrolithiasis and osteoporosis (52). Therefore, dietary patterns may involve the reciprocal association between osteoporosis and nephrolithiasis in the time period.

Nephrolithiasis is thought to be a risk factor for osteoporosis, but data assessing if osteoporosis predisposes to the risk of nephrolithiasis are lacking. In this study, we clarified the two phenomena underlying nephrolithiasis and osteoporosis by summarizing all the data from the relevant studies. To the best of our knowledge, the present study is the first study to quantify the association between nephrolithiasis and the risk of osteoporosis and *vice versa* through a cumulative analysis. Some strengths existed in this study, including large sample size (a total of 454,464 participants), high methodological qualities of the relevant studies, fewer heterogeneities among the included studies, and an evidence-based etiological theory for the association between nephrolithiasis and risk of osteoporosis and *vice versa*. However, some inherent limitations should be noted when interpreting the present results. First, only nine relevant studies were included in the analysis, which may yield biased data. Second, all the included studies were retrospectively designed, which may downgrade the evidence of this cumulative analysis. Third, different geographic regions, sample sizes, mean age, study designs, variable factors, and characteristics of participants among the eligible studies might cause potential heterogeneities. Fourth, higher age, gender, hyperlipidemia, hypertension, diabetes mellitus, hyperuricemia, smoking, and being overweight might be the independent risk factors for nephrolithiasis so as well for osteoporosis. Given the inability to adjust for these potential confounders, it’s not possible to conclude that the causal relationship exists between nephrolithiasis and osteoporosis. Based on these facts, additional high-quality rigorous and prospective cohorts are still warranted to better illustrate the reciprocal correlation between nephrolithiasis and the risk of osteoporosis and *vice versa*.

## Conclusion

The present cumulative analysis provides evidence supporting the reciprocal relationship between nephrolithiasis and the high risk of osteoporosis and *vice versa*. However, the evidence of the causal relationship between nephrolithiasis and osteoporosis is not so strong due to the study design and the confounding factors. Further study of these relationships is still required, given the possibilities to screen for these two conditions if necessary. Our study highlights that regular follow-up assessments are needed when managing patients with nephrolithiasis or osteoporosis, especially for the elderly.

## Data availability statement

The original contributions presented in the study are included in the article/**Supplementary Material**. Further inquiries can be directed to the corresponding author.

## Author contributions

Conceptualization and methodology: SJ and WX. Software and formal analysis: YW and WZ. Writing original draft and editing: SJ, JJ and WX. All authors contributed to the article and approved the submitted version.

## Supplementary material

Supplementary Table 1 PRISMA Checklist.

Supplementary Table 2 Newcastle-Ottawa Scale assessment of the quality of the case-control and cohort studies.
